# Microcystin-LR, a Cyanobacterial Toxin, Induces Changes in the Organization of Membrane Compartments in Arabidopsis

**DOI:** 10.3390/microorganisms11030586

**Published:** 2023-02-25

**Authors:** Gabriella Petra Juhász, Sándor Kéki, Anita Dékány-Adamoczky, Csongor Freytag, Gábor Vasas, Csaba Máthé, Tamás Garda

**Affiliations:** 1 Department of Botany, Faculty of Science and Technology, University of Debrecen, H-4032 Debrecen, Hungary; 2 Juhász-Nagy Pál Doctoral School of Biology and Environmental Sciences, University of Debrecen, H-4032 Debrecen, Hungary; 3 Department of Applied Chemistry, Faculty of Science and Technology, University of Debrecen, H-4032 Debrecen, Hungary; 4 Doctoral School of Chemistry, University of Debrecen, H-4032 Debrecen, Hungary

**Keywords:** cyanobacteria, microcystin-LR, protein phosphatase PP2A and PP1, oxidative stress, tonoplast, vacuolar organization, mitochondrial fusion and fission, chloroplast stromule

## Abstract

To evaluate the effects of the cyanobacterial toxin microcystin-LR (MCY-LR, a protein phosphatase inhibitor) and diquat (DQ, an oxidative stress inducer) on the organization of tonoplast, the effect of MCY-LR on plastid stromule formation and on mitochondria was investigated in wild-type Arabidopsis. Tonoplast was also studied in PP2A catalytic (*c3c4*) and regulatory subunit mutants (*fass-5* and *fass-15*). These novel studies were performed by CLSM microscopy. MCY-LR is produced during cyanobacterial blooms. The organization of tonoplast of PP2A mutants of Arabidopsis is much more sensitive to MCY-LR and DQ treatments than that of wild type. In *c3c4*, *fass-5* and *fass-15*, control and treated plants showed increased vacuole fragmentation that was the strongest when the *fass-5* mutant was treated with MCY-LR. It is assumed that both PP2A/C and B” subunits play an important role in normal formation and function of the tonoplast. In wild-type plants, MCY-LR affects mitochondria. Under the influence of MCY-LR, small, round-shaped mitochondria appeared, while long/fused mitochondria were typical in control plants. Presumably, MCY-LR either inhibits the fusion of mitochondria or induces fission. Consequently, PP2A also plays an important role in the fusion of mitochondria. MCY-LR also increased the frequency of stromules appearing on chloroplasts after 1 h treatments. Along the stromules, signals can be transported between plastids and endoplasmic reticulum. It is probable that they promote a faster response to stress.

## 1. Introduction

Presently, toxic cyanobacterial blooms are a serious environmental problem caused by the massive proliferation of cyanobacteria in surface waters. Blooms are occurring with increased regularity in freshwater, and their intensification is due to numerous biological, physical and chemical factors, including climate change. Although the exact causes of water blooms are still unclear, human impact and climate change have contributed to the recent increase in their occurrence [1,2]. Cyanobacteria are photosynthetic prokaryotes that depend on sunlight, carbon dioxide and various nutrients, such as nitrogen and phosphorus, for growth. Nutrient enrichment (eutrophication) in surface waters and rising temperatures contribute to the proliferation of these organisms [3,4,5]. During water blooms, cyanobacteria can also produce harmful metabolites that pose several health and ecological risks. According to their effect on mammals, cyanotoxins have been divided into neurotoxins, hepatotoxins and dermatotoxins. However, the most common and dangerous cyanotoxins are hepatotoxins, which can cause severe liver damage and appear to be strong liver tumor promoters as protein phosphatase inhibitors [6].

The increasingly present toxins from toxic water blooms can bioaccumulate in aquatic organisms, including in plants [7]. These cyanotoxins present a serious threat to human health because drinking water from contaminated freshwater or consuming organisms that lived in it can harm human health. In addition, many of their effects on plants are also known. Microcystins (MC) are the most abundant of these metabolites, which can be produced by various cyanobacteria such as *Microcystis*, *Anabena*, *Oscillatoria* or *Nostoc*. It has been shown that biosynthesis and extracellular concentration of MC enhanced by exposure to bright light may have an impact on the toxicity of cyanobacterial water blooms [8,9]. Currently, at least 279 microcystin variants have been reported, but MCY-LR is the most frequently occurring and studied form [10].

**Figure 1:**
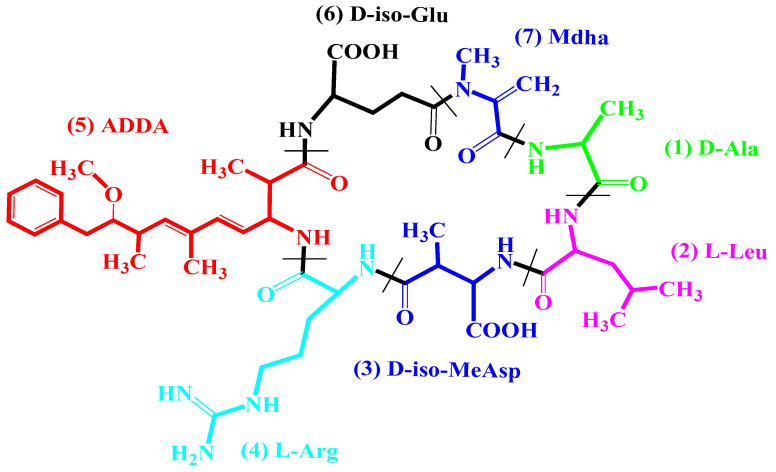
Structural formula of MCY-LR and color coding of amino acids [17].

MCY-LR is a cyclic heptapeptide consisting of seven amino acids (including three D-amino acids, two variable L-amino acids and two unusual amino acids, namely N-methyldehydroalanine (Mdha) and a hydrophobic D-amino acid, 3-amino-9-methoxy-2-6,8-trimethyl-10-phenyldeca-4, 6-dienoic acid (Adda) (Figure 1). Microcystins differ mainly in their two L-amino acid side chains. In the case of MCY-LR, the second amino acid is L-leucine, while the fourth is L-arginine [11,12]. The primary targets of MCY-LR are type 1 and 2A serine/threonine protein phosphatases (PP1 and PP2A) and, therefore, it may interfere with several signaling and metabolic pathways in eukaryotes, including in plant cells. The IC_50_ of MCY-LR has been determined to inhibit protein phosphatase in the concentration range of 0.1–0.25 nM in vitro [13]. The methylene group of MCY-LR MDHA is covalently bound to the catalytic subunit of both PP1 and PP2A, but this is not required for the inhibition of protein phosphatases. The ADDA side chain is responsible for the inhibitory effect as it binds to the active center of protein phosphatases. It is the introduction of Adda into the hydrophobic groove at the catalytic site of protein phosphatase that renders protein phosphatases inactive [14,15,16]. In addition to inhibiting protein phosphatases, MCY-LR also induces the formation of reactive oxygen species (ROS) in both plant and animal cells [8,9].

The plant vacuole is a multifunctional organelle and essential for plant development and growth. It is a highly dynamic structure that is constantly changing [18]. This dynamic involves mainly microfilaments that support vacuolar structure and the acto-myosin pathway that plays a crucial role in vacuolar morphogenesis [19]. In human cells, MCY-LR is known to induce ER stress, mitochondrial damage and autophagy [17]. Many effects of MCY-LR have been reported in plant cells: (1) inhibition of germination, growth and cell elongation; (2) microtubule reorientation; (3) necrosis in shoot and root; (4) division spindle abnormalities; (5) reduction in auxin transport proteins and (6) nuclear fragmentation [20,21,22,23]. However, limited information is known about the effect of protein phosphatase inhibitors on plant endomembrane integrity. MCY-LR induces disorganization of the F-actin network, resulting in the accumulation and reduction of endoplasmic reticulum elements and aggregation of Golgi dictyosomes [24]. Nagy and co-workers observed the fragmentation of tonoplasts and engulfment of plastids in tonoplast-coated vesicles in Arabidopsis hypocotyl cells after short-term MCY-LR treatments [25].

It is assumed that mitochondrial fission is necessary for the inheritance and maintenance of mitochondria. Arabidopsis (*Arabidopsis thaliana*) has well-conserved Dnm1/Drp1 orthologs, DRP3A and DRP3B, which are involved in mitochondrial fission [26,27]. Disruption of these genes results in the formation of elongated mitochondria. Two homologs of the outer-membrane-anchored protein Fis1, AtFis1a (BIGYIN) and AtFis1b, also were found, and disruption of their gene expression in Arabidopsis caused the mitochondria to become larger but no elongation was observed [28,29,30]. It has been established in human and animal models that PP2A/Bβ2 (Protein phosphatase 2A regulatory subunit Bβ2) antagonize survival by inducing Drp1- and Fis1-dependent mitochondrial fragmentation. An external mitochondrial PP2A holoenzyme is a critical regulator of mitochondrial morphogenetic balance that determines the sensitivity of neurons to various types of injury [31]. In animals, MCY-LR treatment induced changes in Fis1 and Drp1 protein levels, which may be associated with MCY-LR-induced mitochondrial dynamic disorder and fission aggravation [32].

Stromules are stroma-filled tubules that extend from all types of plastids. They are highly dynamic structures with various lengths and are less than 1 µM thick. Their function is not fully understood but initially it was thought that they enable the transfer of Rubisco molecules or macromolecules between interconnected plastids [33,34]. Later studies show that this type of macromolecule transfer has a very low probability [35]. Stromules provide a much larger surface area for the transport of metabolic intermediates into plastids and from there to other organelles. A close association of stromules with mitochondria has been observed in *Iris unguicularis* epidermal cells and tobacco hypocotyl cells [36,37]. Strong induction of chloroplast stromules has been demonstrated during immune responses in Arabidopsis and *Nicotiana* plants. Signals present in the initial phase of programmed cell death (PCD), such as salicylic acid or hydrogen peroxide, can cause the appearance of stromules, even with exogenous application of these molecules [38]. Their role is not yet clear, but some studies suggest that stromules may play a role in short-term stress responses [39].

In light of the above, our objectives were as follows: (1) How do protein phosphatase inhibition and ROS induction by MCY-LR affect tonoplast integrity and vacuole organization? (2) How are tonoplast integrity and vacuole organization altered in protein phosphatase catalytic and regulator subunit mutant Arabidopsis plants? (3) How do MCY-LR-induced protein phosphatase inhibition and ROS induction affect mitochondrial fusion/fission? (4) How does MCY-LR affect the stromule frequency of chloroplasts?

## 2. Materials and Methods

### 2.1. Chemicals

1-(2-acryloyloxy-3-chloro-prop-1-yl)-amino-5-isocyanonaphthalene (ACAIN) was synthesized as described in a previous publication [25]. Microcystin-LR (MCY-LR) was purified from *Microcystis aeruginosa* with the same methodology as previously described [40]. Diquat dibromide monohydrate (DQ) was purchased from Sigma-Aldrich.

### 2.2. Plant Material and Treatments

*Arabidopsis thaliana* genotypes used were wild-type Col0 and protein phosphatase (PP2A) mutants *c3c4* (PP2A C double catalytic subunit mutants), E288, N84613 (*fass-5* and *fass-15* mutants for the B” regulatory subunit) [41,42,43]. Mutant seeds were a gift from Dr. Martine Pastuglia, INRA, Versailles, France. Seeds were surface-sterilized by rinsing with 70% ethanol, then 20% of commercial Na-hypoclorite containing disinfectant (Domestos), followed by washing with sterile water. Sterilized seeds were transferred to MS medium (with Gamborg vitamine, Duchefa Biochemie, Haarlem, The Netherlands) supplemented with 2% sucrose (MS*) and 0.9% agar [44,45]. After 48 h of cold treatment, the plates were transferred to a tissue culturing room (photoperiod: 14 h light, 10 h dark, photon flux density: 60 µmol m^−2^ s^−1^, temperature: 22 ± 2 °C) [22].

After 4 days of culturing, parts of the seedlings were treated with MCY-LR and DQ. Aqueous stock solutions of MCY-LR and DQ were diluted to 1 µM (MCY-LR) and 0.5 µM (DQ) final concentrations with liquid MS*. Seedlings were transferred to sterile petri dishes containing sterile filter papers soaked with the MCY-LR and DQ solutions or with liquid MS*, in the case of controls. The plates were returned to the tissue culturing room for 24 h. In the case of *fass-15* and *fass-5* mutants, only homozygous recessive seedlings were treated.

### 2.3. Visualization of Chloroplast Stromules and Mitochondria

Mitochondria and chloroplast stromules were examined by using plants bearing GFP-fusion protein constructs in a Columbia background (kindly provided by Dr. Jaideep Mathur, University of Guelph, ON, Canada) as follows. Both organelles were examined as time-dependent effects of 1 μM MCY-LR.

For the analysis of mitochondria, plants bearing a fusion of the mitochondrial β-ATPase subunit to GFP and a tri-peptide-targeting YFP to the peroxisome were used; thus, peroxisomes were also visualized [46,47]. For visualization, a Leica TCS-SP5 CLSM (a facility given by Dr. Jaideep Mathur, University of Guelph, ON, Canada) was used with the following settings: 488 nm Ar and 543 He–Ne lasers with emission spectra acquired at 503-515 nm for GFP (detection of mitochondria). YFP signals for peroxisomes were detected as merged images of GFP and RFP channels. Mitochondria were analyzed for size and the numbers of hypocotyl cells with predominantly small-sized and, respectively, elongated mitochondria were analyzed and plotted. At least 300 cells were examined per treatment per experiment and experiments were repeated three times per each treatment time (1, 4, 6 and 24 h).

For the analysis of stromule formation, plants bearing stroma-targeted tpFNR:GFP constructs [48] were used. CLSM settings were similar to those of the visualization of mitochondria. Stromule frequencies were calculated as the percentage of plastids bearing stromules at the moment of their observation. The occurrence of multiple stromules per plastid was not analyzed, as we simply followed the occurrence of these stroma extensions. At least 300 cells of Col0 hypocotyls were examined per treatment per experiment and experiments were repeated at least four times for each treatment time (1 h–5 d range).

Data were plotted with the aid of Systat SigmaPlot 12.0 software, and SE values are shown on plots.

### 2.4. ACAIN Staining and Microscopy

Staining of tonoplasts with ACAIN was performed essentially as described previously [25]. The seedlings were placed in 24 well tissue culture plates and washed with phosphate-buffered saline (PBS). After the initial washing, seedlings were treated with 0.025% Triton X-100 (Reanal, Budapest, Hungary) solution for 5 min, followed by a washing step with PBS. ACAIN stock dissolved in DMSO was diluted with PBS to a final concentration of 20 µg mL^−1^ and used for the staining of the seedlings. The duration of the staining was 30 min, followed by 3 × 5 min washes. Finally, the samples were immediately visualized in a Nikon Ti-E inverted super-resolution microscope (Nikon Instruments Inc., Melville, NY, USA) with NIS-elements Ar software. For ACAIN dye visualization we used 405 nm laser, and for the detection of chloroplast autofluorescence we used a 647 nm laser. Hypocotyls of both Col0 and *fass-15*/*fass-5* mutants were analysed for ACAIN staining.

Experiments were repeated three times per each treatment (treatment time was 24 h). The treatment time frame was set up according to [25] to avoid long-lasting treatments that already induce cell death. Data were plotted with the aid of Systat SigmaPlot 12.0 software; SE values are shown on plots.

## 3. Results

### 3.1. Changes in Tonoplast Organization Induced by MCY-LR and DQ in Wild-Type, c3c4, Fass-5 and Fass-15 Mutant Arabidopsis Thaliana (Col-0) Seedlings

**Figure 2:**
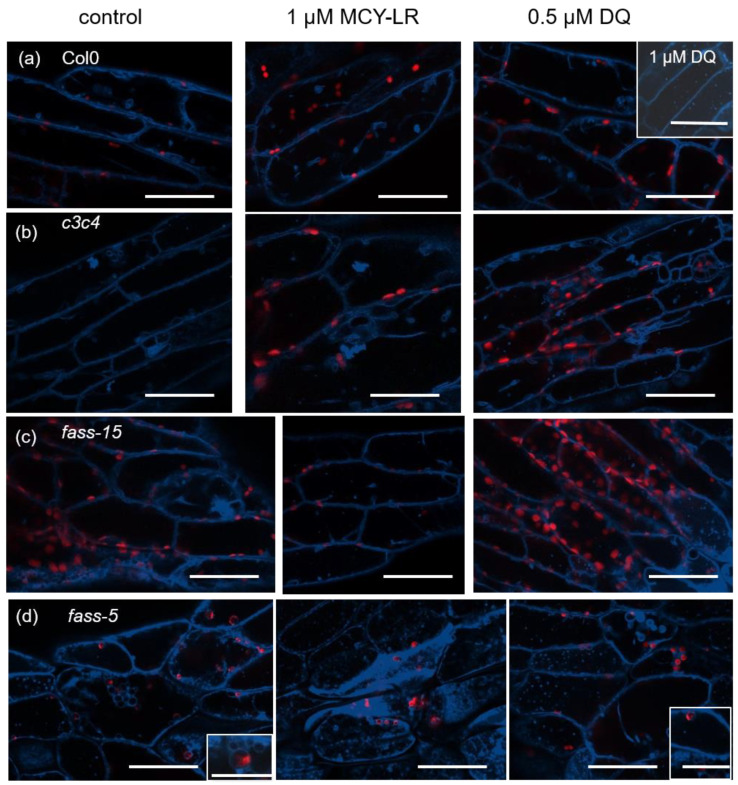
The organization of tonoplast systems of several Arabidopsis PP2A-related mutants is more sensitive to 24 h MCY-LR and DQ treatments than those of wild-type plants. Composite CLSM images of hypocotyls showing chlorophyll autofluorescence (red) and tonoplasts labeled with ACAIN (blue). (**a**) Col0, (**b**) *c3c4*, (**c**) *fass*-*15* and (**d**) *fass-5*. (**a**) In the Col0 wild-type hypocotyls, no visible effect could be detected after toxin treatments in general. Only a high concentration (1 µM) of DQ induced an increase in the occurrence of small tonoplast-coated vesicles (called “vesicularization”). (**b**) In *c3c4*, controls show tonoplast-coated small vesicles. Their occurrence increased in the presence of MCY-LR and DQ induced intense vesicularization. (**c**,**d**). Increase of vesicularization in the *fass* mutants. This is true for controls, and the phenomenon intensified at MCY-LR and DQ treatments. This vesicularization/vacuole fragmentation process was more intense in *fass-5* and culminated at MCY-LR treatment (**d**). Inset picture in (**d**, control) shows a chloroplast entrapped in a tonoplast-coated vesicle. Scale bars: 50 µm.

Live plants were used for our tests, in which the tonoplast (blue) was visualized with ACAIN labeling, and chlorophyll autofluorescence is visible in red. The living plants were needed in order to be able to examine the dynamics of tonoplast under the influence of MCY-LR and DQ. During our studies, we examined the epidermal and cortex cells of hypocotyl. In the case of Col-0 untreated plants, large vacuoles and some smaller tonoplast-coated vesicles were clearly visible (Figure 2a). Col-0 plants treated with 1 µM MCY-LR and 0.5 µM DQ did not show abnormal tonoplast organization after 24 h of treatment. Only a high concentration of DQ (1 µM) increased the occurrence of small tonoplast-coated vesicles (Figure 2a). Minimal vesicularization (that is, vacuole fragmentation) was observed in untreated plants of the double catalytic subunit mutant *c3c4*. In *c3c4* plants, 1 µM MCY-LR induced a small increase in vesicularization, whereas 0.5 µM DQ induced a drastic increase (Figure 2b). In *fass-15* and *fass-5* mutants, intense vesicularization was observed in untreated plants (Figure 2c,d). In fass-15 plants, fragmentation of vacuoles to small vesicles was slightly intensified in the presence of both 1 µM MCY-LR and 0.5 µM DQ, but typically a more drastic change occurred in response to DQ, characterized by a higher frequency of small tonoplast-coated vesicles (Figure 2c). The *fass-5* mutant plants showed the most drastic changes in tonoplast organization. The intense vesicularization of the cells can be observed, which is also characteristic of untreated plants but reaches its maximum in the case of 1 µM MCY-LR treatment. In the cells of *fass-5* plants, fragmentation of vacuoles and chloroplasts entrapped in tonoplast-coated vesicles were also observed in treated and untreated plants (Figure 2d).

We also investigated the dynamics of the vacuolar system under the effects of MCY-LR and DQ in wild-type Col-0, *c3c4*, *fass-5* and *fass-15* mutant Arabidopsis plants. No abnormalities were found in the vacuolar dynamics of Col-0 plants after 24 h of treatment with either MCY-LR or DQ (Supplementary Movie S1). For the *c3c4* and *fass-15* Arabidopsis mutants, vacuolar dynamics were similar to those of control plants, with no significant change observed in response to treatments (Supplementary Movie S2). For the *fass-5* regulator subunit mutant, no vacuolar dynamics or slow movement were observed in untreated plants. However, in MCY-LR or DQ treatment of these plants, vacuolar dynamics were completely inhibited (Supplementary Movie S3).

### 3.2. Effect of MCY-LR on Mitochondrial Morphology

To study mitochondria, we used “mito-GFP” + YFP-PTS1 with Col-0 background. As for the study of tonoplasts, we examined the hypocotyl cells of seedlings. The duration of the study took place in the time range of 1–24 h. Mitochondria move dynamically within the cell and their shape changes depending on the developmental stage and physiological conditions of the cell. Mitochondrial morphology is regulated by a dynamic balance between fission and fusion [49].

**Figure 3:**
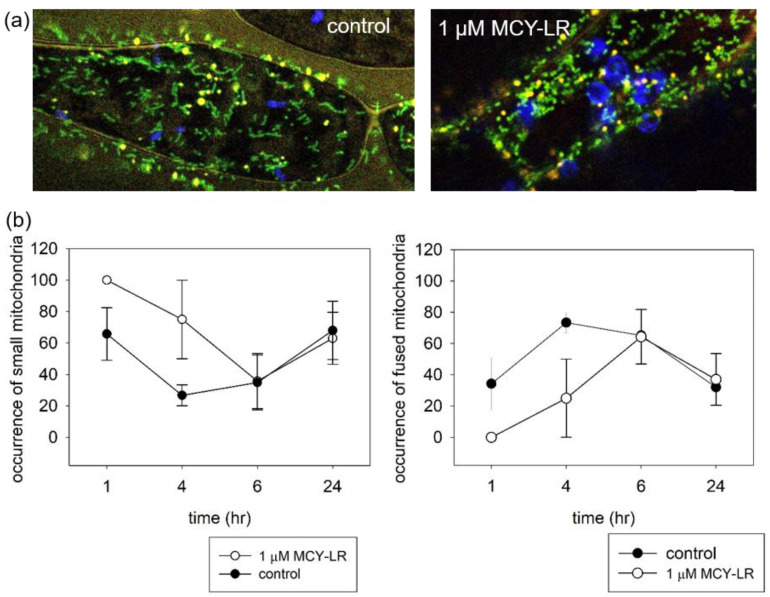
Short-term (1–4 h) MCY-LR treatment induces mitochondrial fission or inhibits fusion. (**a**) CLSM microscopic images of hypocotyl cells showing mitochondria (green), peroxisomes (yellow) and chloroplasts (chlorophyll autofluorescence, pseudo-colored in blue). Control cells show mainly long/fused mitochondria, while in the presence of 1 µM MCY-LR (1 h exposure), mostly small, round-shaped mitochondria occur. Scalebars: 10 μm. (**b**) Time-course of the frequency of small and fused mitochondria, respectively.

Mitochondria in cells of untreated plants show a diverse morphology. Mostly elongated (rod-like) fused mitochondria are typical, but small round mitochondria can also be observed. After short-term MCY-LR treatment, the rod-like mitochondria disappeared, and only relatively small spherical mitochondria were characteristic (Figure 3a). The dynamic change in mitochondrial morphology can be observed in Figure 3b. After a short term (1–4 h) treatment, 1 µM MCY-LR modified this dynamic until it returned to control levels after 6 h. MCY-LR treatment (1–4 h) clearly decreased the number of rod-like fused mitochondria and increased the number of small spherical mitochondria (Figure 3b). MCY-LR inhibited PP1 and PP2A activity and did not affect ROS levels during 4 h of treatment in hypocotyls (to be published elsewhere).

### 3.3. Effect of MCY-LR on Chloroplast Stromule Formation

The dynamics of stromules are very rapid and variable (length, diameter). The appearance and frequency of stromules also depends on the type of cells and the size of the plastids. The tests were carried out over several days using tpFNR:GFP plants with hypocotyl cells.

**Figure 4:**
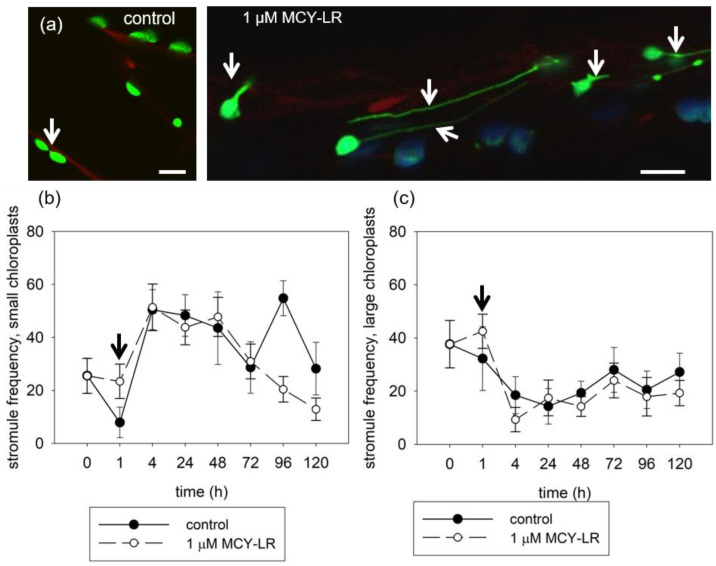
MCY-LR increases stromule frequency at a short term of exposure and decreases it at long exposures. (**a**) For small-sized chloroplasts in hypocotyls, 1 h of MCY-LR treatment of tpFNR:GFP plants shows a visible increase of stromule occurrence (arrows). Scalebar 10 µM. (**b**,**c**) Time-dependent effects of MCY-LR on stromule frequencies for small (**b**) and large (**c**) chloroplasts. Arrows show increases of stromule frequencies at 1 h MCY-LR exposures.

In the untreated plants, a relatively small number of stromules were observed and they were short. A stimulation of stromule formation was observed on chloroplasts after 1 h of treatment using 1 µM MCY-LR. These stromules were extremely long (Figure 4a). The frequency of stromules increased only after short-term (1 h) MCY-LR treatment, compared with that of the control. This phenomenon was observed for both small and large chloroplasts. At all other examined time points, the frequency of stromules did not differ from untreated plants in large chloroplasts and decreased in small chloroplasts after 4–5 day treatments (Figure 4b,c).

## 4. Discussion

The toxins produced by cyanobacteria pose a serious risk to living organisms, including humans, animals and plants. The mechanism of MCY toxicity is mainly the inhibition of protein phosphatases of type 1 and 2A (PP1 and PP2A), and it induces the formation of reactive oxygen species [8,9]. There have been several studies on the subcellular changes caused by MCY-LR but very little research on the effects of the toxin on the vacuolar system, mitochondria and chloroplasts in plants.

The stromule is a highly dynamic structure that can be observed in all types of plastids. These dynamic structures are constantly present in cells, but their number in a given moment can be is low. We also observed low stromule frequency in the hypocotyl cells of untreated Arabidopsis plants (Figure 4a). The increase of stromule formation was observed during short-term (1 h) treatments with 1 µM MCY-LR (Figure 4a), which may be a response to the increase in the ROS level induced by MCY-LR, since inhibition of protein phosphatases (PP1 and PP2A) by MCY-LR was only detectable at 4 h of treatment (data not shown). Nevertheless, it is more likely that the increase in frequency of stromules was part of a general stress response to the presence of MCY-LR. The effect of MCY-LR, an increase in stromule frequency, was observed only at 1 h of treatment for both small and large chloroplasts (Figure 4b,c).

Mitochondria go through a continuous cycle of fission and fusion. Fission is a process independent of the regular division of mitochondria since it does not involve DNA replication. Together, these two processes contribute to the regulation of the number, size and shape of mitochondria in cells [50]. We observed diversity in the shapes of mitochondria in untreated Arabidopsis hypocotyl cells. Both rod-like and small spherical mitochondria were characteristic of the cells (Figure 3a). DRP3A and DRP3B are involved in mitochondrial fission and localize to the mitochondrial fission site. In their absence, elongated, networked mitochondria were formed. Disruption of gene expression of AtFis1a and AtFis1b caused mitochondrial enlargement. Changes in mitochondrial morphology were observed upon short-term (1–4 h) treatment with 1 µM MCY-LR. Treatment was characterized by enlarged spherical mitochondria and the disappearance of elongated mitochondria. Overexpression of FIS1A and FIS1B was associated with increased mitochondrial fission. In COS-7 human cells, fragmentation of mitochondria has also been reported in the presence of myc-hFIS1 overexpression [51,52]. In addition, a mitochondrial membrane-bound ubiquitin protease (UBP27) contributes to mitochondrial morphogenesis in Arabidopsis. Upon overexpression of UBP27, mitochondrial morphology changed from rod-shaped to spherical in a way similar to during overexpression of DRP3 [53]. Based on these findings, it is hypothesized that MCY-LR may affect the activity of these proteins, thus resulting in the spherical mitochondrial morphology. In addition, morphological changes in mitochondria have been shown to occur in response to various stresses. Yoshinaga et al. [54] detected morphological changes in mitochondria upon H_2_O_2_ and paraquat treatment, where the normal rod-like shape was transformed into a spherical form, and a similar phenomenon was observed upon inhibition of the myosin ATPase. Similar mitochondrial morphological changes were also observed with methyl viologen (paraquat, ROS inducer) and heat shock in Arabidopsis mesophyll protoplast [55].

Live plants were used to investigate the tonoplasts by examining vacuolar dynamics under the effect of a protein phosphatase inhibitor and ROS-inducing cyanotoxin (MCY-LR) and an ROS-inducing herbicide (DQ, used here as a reference for oxidative stress induction). The tonoplast was visualized with ACAIN, which does not alter the dynamics and organization of the tonoplast [25]. In untreated wild-type Col-0 plants, large vacuoles and some tonoplast-coated vesicles were observed (Figure 2a) and vacuolar dynamics were normal (Supplementary Movie S1). Disorganization of the tonoplast and chloroplasts entrapped in tonoplast-coated vesicles were observed after 24 h of treatment with 5 µM MCY-LR, while, in the case of 1 µM MCY-LR, several tonoplast-coated vesicles were observed after 4 h of treatment [25]. After 24 h of treatment with 1 µM MCY-LR and 0.5 µM DQ, no abnormalities in tonoplast organization or vacuolar dynamics were found in wild-type plants (Figure 2a, Supplementary Movie S1). Our results may be explained by the fact that after 24 h, the tonoplast organization and dynamics of Col-0 plants were restored and the effect of much a lower toxin concentration is probably not as strong. However, 1 µM DQ treatment increased the frequency of tonoplast-coated vesicles (Figure 2a, inset image). Higaki et al. [19] observed a close association between the vacuolar membrane and actin microfilaments (MFs), where actin was localized on the surface of the vacuoles and at the periphery of the cytoplasmic strands of the vacuoles. The dynamics of actin and the cytoplasmic strands between vacuoles also overlapped. Thus, this demonstrates that MFs play an important role in vacuolar morphology [19]. In *c3c4* mutant Arabidopsis plants, we observed vesicularization in untreated and MCY-LR-treated plants, which was intensified by the toxin (Figure 2b). In the *fass-5* and *fass-15* mutant plants, this vesicularization was even more intense in untreated plants and was enhanced by MCY-LR treatment (Figure 2c,d). The vesicularization/vacuole fragmentation process is more intense in *fass-5* and culminates at MCY-LR treatment. Furthermore, chloroplasts entrapped in tonoplast-coated vesicles in *fass-5* mutant Arabidopsis were observed at MCY-LR and DQ treatment and also in control plants (Figure 2d, inset image). We suggest that these vesicularisation processes may be due to polymerization disorder and over-bundling of actin microfilaments. Higaki and co-workers observed synchronous shrinkage then disappearance of MF and vacuolar cytoplasmic strands, resulting in small spherical vesicles at the end, in response to bistheonelide A (BA, an actin polymerization inhibitor). These observations are similar to the vesicularization we found in *c3c4* and *fass* mutants, which was intensified by MCY-LR. The effect of MCY-LR on actin microfilaments has also been described regarding *Oryza sativa* root cells. Strong bundling of actin microfilaments and actin stabilization (lack of dynamics) due to a lack of dephosphorylation induced by MCY-LR have been described, which was associated with the inhibition of protein phosphatase by the toxin in rice [24]. However, we did not detect any visible disorders in the MF system in Arabidopsis hypocotyls (data not shown), and Arabidopsis *crk* or *dis* mutants impaired in actin organization did not show notable changes in vacuole organization and dynamics (Supplementary Movie S4). For MCY-LR treatments, vacuole fragmentation is rather a result of the induction of cell death processes that do not involve MF organization, as suggested before [25]. It should be noted that PP2A inhibition by MCY-LR was observed in Col-0 plants after 24 h of treatment, which was more intense in catalytic subunit mutants, but MCY-LR had more complex effects in *fass* mutants (to be published elsewhere).

We observed a similar vesicularization process and vacuolar fragmentation in the effects of ROS-inducing DQ treatments as in MCY-LR treatment. These effects were common to both *c3c4* and the two *fass* mutants (Figure 2b–d). However, in the case of *fass-5* mutant (untreated, MCY-LR and DQ treatment), we observed chloroplasts entrapped in tonoplast-coated vesicles, which was not characteristic of Col-0, *c3c4* and *fass-15* mutants. Like MCY-LR, DQ also amplified the abnormalities observed in control plants. The *fass-5* mutant was the most sensitive to DQ-induced oxidative stress, according to our results (Figure 2d). Since DQ does not induce protein phosphatase inhibition in *Arabidopsis thaliana*, oxidative stress is probably responsible for vesicularization and vacuole fragmentation. In *c3c4* and *fass* mutants, strong oxidative stress was described in untreated plants, which was enhanced by DQ, whereas no increase in ROS levels was observed in Col-0 plants in effect of DQ [56]. An extensive, well-organized, dynamic network of actin microfilaments has been reported in Arabidopsis hypocotyl cells. Upon treatment with paraquat (PQ, which induces oxidative stress similar to DQ), massive bundling of actin microfilaments, a decrease in actin microfilaments density and reduced actin dynamics were observed [57]. These data suggest that structural and dynamic changes in MF may also underlie the tonoplast abnormalities found upon DQ treatment. Based on these data, the tonoplast abnormalities after DQ treatment may also be due to structural and dynamic changes in MF.

## 5. Conclusions

Our results contribute to the understanding of the harmful effects of MCY-LR, a microbial toxin in vascular plants. The effects of this toxin are complex and interfere with many cellular processes. Our main findings were as follows: (i) MCY-LR induces changes in tonoplast organization and dynamics. Studies on the PP2A-related mutants reveal that these effects can be at least partially attributed to alterations in PP2A functionality. DQ, an inducer of oxidative stress, can also promote vacuolar fragmentation. (ii) MCY-LR affects the fission–fusion of mitochondria and modifies chloroplast stromule frequency. We suggest that these are stress reactions, partially as consequences of PP2A inhibition. Such studies contribute to the understanding of the harmful effects of this cyanotoxin in model plants, but they can also be extended to important crops.

## Data Availability

Data will be made available on request.

## Supplementary Materials

The following supporting information can be downloaded at: https://www.mdpi.com/article/10.3390/microorganisms11030586/s1, Movie S1: Vacuole dynamics in hypocotyl cells of Col0 control plant. Blue: ACAIN labeling, red: chlorophyll autofluorescence, Movie S2: Vacuole dynamics in hypocotyl cells of *c3c4* mutant/control (untreated) plant. Blue: ACAIN labeling, red: chlorophyll autofluorescence, Movie S3: Vacuole dynamics in hypocotyl cells of *fass-5* mutant/control plant. Blue: ACAIN labeling, red: chlorophyll autofluorescence, Movie S4: Vacuole dynamics in hypocotyl cells of *crk* mutant/control plant. Blue: ACAIN labeling.
